# Effects of Age and Rice Straw Inclusion Levels in the Diet of Yiling Cull Cows on Growth Performance, Meat Quality, and Antioxidant Status of Tissues

**DOI:** 10.3390/ani11061732

**Published:** 2021-06-10

**Authors:** Xinjun Qiu, Xiaoli Qin, Liming Chen, Qinghua Qiu, Haibo Wang, Muhammad Aziz ur Rahmanand, Binghai Cao, Huawei Su

**Affiliations:** 1 State Key Laboratory of Animal Nutrition, College of Animal Science and Technology, China Agricultural University, Beijing 100193, China; qiuxinjun@cau.edu.cn (X.Q.); qinxiaolicau@outlook.com (X.Q.); rcauqqh@cau.edu.cn (Q.Q.); harper.wang@cau.edu.cn (H.W.); 2 Hubei Fulljoywo Agricultural Development Company Limited, Yichang 443200, China; clm191213@163.com; 3 Institute of Animal and Dairy Sciences, University of Agriculture, Faisalabad 35200, Pakistan; drazizurrahman@uaf.edu.pk

**Keywords:** age, antioxidant status, straw levels, meat quality, Yiling cull cows

## Abstract

**Simple Summary:**

Several negative attributes are associated with carcass and meat from cull cows, including lower carcass weights, inferior tenderness, etc. These undesirable characteristics are normally more distinct with increasing age. In addition, there are no recommended standards for forage levels in the diet of Chinese local cattle to produce high marbled beef. In the current study, we evaluated the effects of age and forage levels on the finishing performance of Yiling cull cows under high-energy ration conditions, and also investigated their antioxidant status related to age and diet. We found that younger age and adequate forage had better growth performance and carcass traits. Our findings highlight the importance of selecting according to age and providing adequate forage feed for Yiling cull cows. Moreover, our study also demonstrated the excellent capability of producing high marbled beef for Yiling cattle.

**Abstract:**

The objectives of this study were to investigate the effects of age and dietary straw levels on growth performance, carcass and meat traits, as well as tissue antioxidant status of Yiling cull cows. Twenty-four Yiling cull cows were arranged in a 2 × 2 factorial design: two age classes consisting of younger cull cows (YCC; appearing with three or four pairs of permanent teeth) and older cull cows (OCC; worn out teeth); two dietary treatments consisting of lower and higher rice straw levels (LRS and HRS; providing 0.7 kg/d and 1.2 kg/d rice straw per head based on air-dry basis, respectively). Cows were fed twice a day. Straw was offered at half of the predetermined weight each meal; concentrate was separately supplied ad libitum. After 300 d of feeding, final body weight (BW), total BW gain, average daily gain and gain:feed intake were higher (*p* < 0.01) in the YCC group than in the OCC group. Total dry matter intake was higher (*p* = 0.03) in the HRS group than in the LRS group, but neutral detergent fiber apparent digestibility was negatively affected (*p* = 0.01) by increased straw levels. Decreased C15:0, C17:0, C20:5n3c, and saturated fatty acids (SFAs) proportion as well as increased C18:1n9c and monounsaturated fatty acids (MUFAs) proportion in meat from YCC with HRS diet were observed as compared to that in meat from YCC with LRS diet (*p* < 0.05). Meat from HRS group had higher (*p* = 0.04) C18:3n3c proportion than meat from LRS group. No significant differences (*p* > 0.05) were found for meat quality attributes except for cooking loss, which was higher (*p* = 0.02) in the HRS group than in the LRS group. Both YCC group and HRS group had higher (*p* < 0.05) cold carcass weight compared to OCC group and LRS group. Moreover, catalase activity of liver tissue was higher (*p* = 0.045) in YCC than in OCC, while superoxide dismutase activity of muscle tissue was higher (*p* = 0.01) in LRS than in HRS. Based on results, we concluded that younger age and feeding high-level straw can improve the finishing performance of Yiling cull cows.

## 1. Introduction

In beef meat production systems, carcass and meat traits depend on breed, age, and diets of slaughtered animals. Previous studies have reported that the tenderness of meat has a negative correlation with the age of cull cows [1,2] due to the chemical nature of connective tissue [3]. Furthermore, negative attributes associated with carcass and meat from cull cows, including lower carcass weights, smaller *Longissimus dorsi* muscle (LM) areas, inferior muscling, yellower external fat color, and darker lean color [4,5]. Generally, providing high-energy ration to cull cows before slaughter improves carcass characteristics and meat quality [6,7] and reverses such an influence.

The inclusion and the type of forage in the diet of finishing cattle depend upon the availability, price, and its influence on fattening and meat quality [8]. Providing forage in sufficient amounts is considered as the key factor in preventing ruminal acidosis due to increased saliva production [9]. However, excessive amounts of forage tend to reduce dry matter intake (DMI), which may be related to its big bulk, slow rumen passage rate and low apparent digestibility [10,11] and consequently influence finishing performance. Therefore, the appropriate amounts of forage are required to maintain rumen health and maximize finishing performance.

In high marbled beef production system, especially with cull cows, the study on the effect of age and forage levels in the diet could be of great significance. In previous studies with cull cows, few experiments have been conducted dealing with the effect of the breed [1,12], finishing diet [13,14], and age [1,2]. They have shown that breed and interaction with finishing diet can modify the fat deposition in the carcass and meat, the muscle fiber type, the collagen content and composition, and in consequence the meat quality in terms of tenderness, flavor, and juiciness. Furthermore, few experiments have studied the relationships of different types of finishing diets with beef meat quality of heifers [15], young bulls [16], and cull beef cows [17], but they did not study factors such as age and their interaction with finishing diet.

Yiling cattle originated in Yichang City and has been identified as a new breed of Chinese yellow cattle. Its history as a draft animal can be traced back to 4000 years. Yiling cattle is currently considered as a draught and meat type breed. Previous studies have evaluated the genetic background of Yiling cattle [18], but the information about the finishing performance and meat traits of Yiling cattle is still partially unclear.

Antioxidant systems are an important determinant of the oxidative stability of stored meat, influenced by breed, age, diet, and environment. The aging process is caused by deleterious effects of reactive oxygen species (ROS) generated spontaneously from normal cellular metabolism [19]. In beef production, cull cows had higher myoglobin or heme-Fe contents [20], which accelerates the oxidation process [21]. Previous study elaborated that inclusion of forage in the diet of beef cattle during the finishing phase altered antioxidant status [22]. However, the antioxidative status of tissues (i.e., liver and muscle tissues) in cull cows in the finishing phase concerning animal age is still needed to be investigated.

For these reasons, our objectives were to assess the effects of age and rice straw inclusion levels in the diet of Yiling cattle on carcass characteristics, and intramuscular fat (IMF) content, tenderness and water-holding capacity of meat. In the current study, we hypothesized that the reduction of the amounts of straw in the finishing diet of cows would show better beef quality due to the potential enhancement of energy intake. Besides, younger cull cows may have greater finishing performance due to their higher eating appetite and capacity. The other objective of the current study was to evaluate the tissue antioxidant status of Yiling cull cows. We hypothesized that older age would induce protein and lipid oxidation of tissues and consequently enhance its antioxidant capacity. Moreover, dietary forage levels may alter antioxidant status of cull cows under high-energy ration conditions.

## 2. Materials and Methods

### 2.1. Animals, Management, and Sampling

All experimental procedures were approved by the Animal Welfare and Ethics Committee of China Agricultural University (Permit No. DK1008). Twenty-four Chinese Yiling cows weighing 330.6 ± 21.1 kg, culled from National Livestock and Poultry Genetic Resources Conservation Farm (Hubei Fulljoywo Agricultural Development Co. Ltd., Yichang, China), were divided into four groups of six animals each in a 2 × 2 factorial design: two age classes consisting of younger cull cows (YCC, appearing with three or four pairs of permanent teeth) and older cull cows (OCC, worn out teeth); two dietary treatments consisting of lower and higher rice straw levels (LRS and HRS; providing 0.7 kg/d and 1.2 kg/d rice straw per head based on air-dry basis, respectively). All cows were reared in separate pens with ad libitum access to concentrate and water. Feeding frequency of concentrate and rice straw were twice a day at 08:00 h and 16:00 h. Rice straw was offered at half of the predetermined weight each meal; concentrate was separately supplied based on 5 to 10% orts. The nutrient composition of concentrate and rice straw are shown in Table 1.

Feed offered and refusals weight were recorded daily to calculate the intake during the whole finishing phases. Concentrate and straw samples were collected every month, and faeces were sampled from the rectum daily at 6:00, 12:00, 18:00, and 24:00 h during the last three days of the test. Samples were dried at 65 °C and smashed by using a mill (Wiley, A. H. Thomas Co., Philadelphia, PA, USA) with a 40-mesh screen, and then stored at −20 °C until further chemical analysis.

Animals were weighed and slaughtered at the 300 d of trial. After evisceration, liver samples were immediately collected from its diaphragmatic surface. Meanwhile, muscle samples were taken with a knife in the LM from the left half carcass between the sixth and seventh ribs. All fresh tissue sample was capped in a tube and stored at −80 °C for the determination of antioxidant status. In the same place, the pH value of carcass was recorded using a Testo 205 pH probe (Testo SE & Co., KGaA, Lenzkirch, BW, Germany) before and after aging for seven days at 4 °C. The hot carcass weight and cold carcass weight (CCW) were recorded to calculate the dressing percentage and the carcass composition, respectively. After aging, approximately 10-cm-thick LM samples were removed from the left half-carcass between the 12th and 13th ribs, and a 5-cm-thick LM sample was immediately separated for physical analysis; the other 5-cm-thick LM sample was vacuum packed and stored at −20 °C for chemical analysis and fatty acids analysis. The LM area between the 6th and 7th as well as between the 12th and 13th ribs were measured using a vegetable parchment with standard grid. The high rib, ribeye, striploin, and tenderloin are considered as the top-grade cuts in China [23]. After cutting, deboning, and trimming, all beef fat and cuts were weighed to calculate the percentage of fat and meat in CCW.

### 2.2. Chemical Compositions

The dry matter (DM, method 934.01), crude protein (CP, method 990.03), and ether extract (EE, method 920.39) and ash (method 924.05) were determined according to the methods of the Association of Offical Analytical Chemists (AOAC 2002) [24]. The organic matter (OM) was calculated as the difference between DM and ash. Neutral detergent fiber (NDF) and acid detergent fiber (ADF) contents were analyzed following the methods of Van Soest et al. (1991) [25]. Chemical analyses were performed on each sample in duplicate. Apparent digestibility was calculated following the endogenous tracer acid-insoluble ash (AIA) method described in our previous trial [26].

### 2.3. Meat Quality

External fat and thick connective tissue were removed and then the LM samples were freeze dried in a freeze-drier (FD-1-50, Biocool, Beijing, China) for 7 days at −50 °C to determine the DM contents. The freeze-dried samples were analyzed for CP and EE according to the AOAC methods [24]. Cooking loss was determined by weight loss during the immersing LM sample (3 × 3 × 3 cm) in a resealable pack bag in a water bath at 80°C until its internal temperature reached 70 °C. Six meat cores (1.27-cm-diameters) parallel to the muscle fiber were removed from each cooked sample, and sheared in the center perpendicular to the muscle fibers using a texture analyzer (TA.XT plus, Godalming, Surrey, UK) for measurement of the Warner-Bratzler shear force (WBSF). Three meat samples (2 × 4 × 1 cm) were pressed using the texture analyzer under 25 kg pressure for 5 min to measure the pressing loss. Drip loss was measured by the weight loss during the suspension of three standardized LM sample (2 × 3 × 5 cm) in a foam box at 4 °C for 24 h.

### 2.4. Fatty Acid Profile

Fatty acid profile in the meat was measured according to the fatty acid methyl ester (FAME) analysis method described in our previous trial [27] with slight modification in internal standard matter. This involved the combination of 100 mg freeze-dried and ground meat with 4 mL of a mixed solution of ethyl chloride and methanol (1:10; *v*/*v*) and 2 mL of an internal standard hexane solution (nonadecanoic acid, 1 mg/mL). After hydrolyzation, neutralization (4 mL of 100 g/L potassium carbonate solution), centrifugation (3000× *g* for 10 min) and filtration (0.2 μm pore size), FAME profile in the supernatant was analyzed using a gas chromatograph (GC-2014, Shimadzu Corporation, Kyoto, Japan) installed with a hydrogen flame detector and a 100 m × 0.25 mm inner diameter × 0.20 μm film thickness capillary column (HP-88; Agilent Technologies, Santa Clara, CA, USA). Nitrogen was used as carrier gas at a flow rate of 1 mL/min and the splitting ratio was controlled at 1:40. The initial oven temperature was 140 °C and held for 5 min, then increased to 230 °C at 3.5 °C/min and held for 15 min. The injector and detector temperatures were set at 280 °C. The injection volume was 1 μL. The chromatographic peak was identified by comparing the relative retention times with those of the standard mixtures of 37 FAME (18919-1AMP, Sigma Chemical Co., Shanghai, China). The relative proportions were calculated as percentages of summed peak areas.

### 2.5. Antioxidant Status

Muscle and liver sample was homogenized with saline solution to determine the concentrations of total antioxidant capacity (T-AOC), catalase (CAT), superoxide dismutase (SOD), malondialdehyde (MDA), protein carbonyl (PC), and ROS. All antioxidant parameters were examined in triplicate using commercial test kits (Nanjing Jian Chen Institute of Biological Technology, Nanjing, China) except for ROS produced by Shanghai Mlbio Institute of Biological Technology (Shanghai, China).

### 2.6. Statistical Analysis

All statistical analyses were carried out using the GLM procedure (SAS Inst. Inc., Cary, NC, USA). The model included the fixed effects of age (YCC and OCC), rice straw levels (LRS and HRS), and the interaction between age and rice straw levels. Treatment means were determined using the LSMEANS option and separated using F-test protected LSD (*p* ≤ 0.05). Differences were considered statistically significant when *p* ≤ 0.05, while 0.05 < *p* ≤ 0.10 was identified as a tendency.

## 3. Results

### 3.1. Growth Performance

The effects of age and straw levels on total intake, apparent digestibility of nutrients and body weight results are shown in Table 2. Initial body weight was similar for the experimental animals. Final BW, total BW gain, average daily gain (ADG) and gain:feed intake (G:F) were higher (*p* < 0.05) in the YCC group than in the OCC group. Total digestible nutrients (TDN) intake, final BW and ADG were tended to be higher (*p* < 0.10) in the HRS group than in the LRS group, but the HRS group had slightly lower (*p* = 0.06) OM apparent digestibility than the LRS group. Total DMI was higher (*p* = 0.03) in the HRS group than in the LRS group. However, the NDF apparent digestibility was negatively affected (*p* = 0.01) by increased straw.

### 3.2. Carcass Characteristics

The effects of age and straw levels on carcass characteristics of Yiling cull cows are shown in Table 3. Both YCC group and HRS group had higher (*p* < 0.01) CCW compared to the OCC group and the LRS group. No significant (*p* > 0.05) differences were observed between both age classes and straw levels for dressing percentage and carcass composition. The area of LM between the 6th and 7th as well as between the 12th and 13th ribs were not affected (*p* > 0.05) by straw levels. Both age and diet had no effect (*p* > 0.05) on the LM area between the 6th and 7th ribs, but the LM area between the 12th and 13th ribs tended to be higher (*p* = 0.07) in YCC than in OCC. High rib and tenderloin were heavier (*p* < 0.05) in the YCC group than in the OCC group, and striploin weight in the HRS group was greater (*p* = 0.02) than in the LRS group.

### 3.3. Meat Quality

The pH value at days 0 and 7 of aging, DM, intramuscular fat (IMF), CP, WBSF, drip loss and pressing loss of the LM from cull cows were not different (*p* > 0.05) between experimental groups (Table 4). Cooking loss was higher (*p* = 0.02) in the LRS group than in the HRS group, but not affected (*p* > 0.05) by age.

### 3.4. Fatty Acid Profile

Fatty acid profile results are in Table 5. The interactions between age and straw levels (*p* < 0.05) were observed for the proportions of C15:0, C16:0, C17:0, C18:1n9c, C20:5n3c, SFAs, and MUFAs. Decreased C15:0, C17:0, C20:5n3c, and SFAs proportion as well as increased C18:1n9c and MUFAs proportion were observed in YCC with HRS diet as compared to YCC with LRS diet (*p* < 0.05). The proportion of C16:0 in YCC with HRS diet tended to be higher (*p* = 0.09) than that in YCC with the LRS diet, but there were no differences (*p* > 0.05) in older cows between the diet groups. The proportion of C18:3n3c was higher (*p* = 0.04) in the HRS group than in the LRS group.

### 3.5. Antioxidant Status

Antioxidant status results showed that T-AOC, ROS, MDA, and PC of both liver and muscle tissue were not different (*p* > 0.05) between the experimental groups (Table 6). The CAT activity of liver tissue was higher (*p* = 0.045) in the YCC group than in the OCC group, but was unaffected (*p* > 0.05) by straw levels. Moreover, the SOD activity of muscle tissue was higher (*p* = 0.01) in the LRS group than in the HRS group, but similar (*p* > 0.05) between age groups.

## 4. Discussion

One of the most important parameters of beef production is the growth ability of animals. Growth in young cattle is unequal and influenced by age and diet. In the current study, younger cows showed higher final BW, total BW gain, and average daily gain, which is supported by the study of Sawyer et al. (2004) [28], who found that DMI and ADG decreased linearly with the increased age of the cull cows. However, the results in growth performance are opposite to the finding of Galli et al. (2008) [29], who reported that younger cows finished under grazing conditions had lower the final BW than older cows. The increased DMI with forage inclusion levels in the diet could be supported by Galyean and Defoor (2003) [30]. They reported a positive linear relationship (*R*^2^ = 0.92) between forage NDF and DMI (% BW), and also claimed that rumen and gut fill does not limit intake when ruminants are fed high concentrate diets and the mechanism for enhanced intake is energy dilution and to maintain energy intake. However, we believe the changed intake is more likely due to the more balanced ruminal environment and the greater ruminal pH value, particularly in high concentrate diet, caused by enhanced chewing activity and saliva flow with increasing forage inclusion [7]. In the present study, calculated by the proportions of total concentrate and straw intake in total DMI during finishing period, the percentages of forage in diet groups were 15.2% and 21.7% (LRS vs. HRS), respectively. Hales et al. (2013) [31] compared different levels of forage inclusion (2%, 6%, 10%, or 14% of alfalfa hay) and showed a quadratic effect of increasing forage proportion on DMI during the whole finishing period in steers. They further reported that an increase in DMI with alfalfa hay inclusion of up to 10%, and then a decrease in DMI with 14% alfalfa hay. A previous study by Swanson et al. (2017) [32] reported that ADG and DMI decreased linearly with increasing forage inclusion. The inconsistency among studies may be due to the differences in breed or forage type. The apparent digestibility of OM (slightly) and NDF decreased with straw levels, which is consistent with the result of Salinas-Chavira et al. (2013) [33]. Generally, Chinese south native cattle were used as draft animals and had not undergone long-term commercial selection. Thus, we speculated that Yiling cattle may be intolerant to forage restriction diet that could cause intake inhibition.

In terms of feed intake, daily gain and feed conversion efficiency, this fattening strategy seems less efficient in resource utilization. The inefficiency may be mainly due to the fact that the experimental cattle were mature cows and the increase in BW comes from fat deposition rather than the growth and development of bones and muscles. Although there is no control group (no fattening) in the current study, the production experience of the farm has proved that long-term fattening can increase the marbling richness of beef from Yiling cull cows. High marbled beef, especially from native cattle, can fetch high prices at the market, which can support the reasonableness of this fattening strategy.

Younger cull cows and cows fed higher straw levels had heavier carcass that could be justified by their higher final BW. A recent study reported that the proportion of fat in carcasses increased, and the relative proportion of muscles decreased as animals become older [34]. However, fat percentage in carcass was similar between age groups in the current study. Dressing percentage is an important indicator in the evaluation of carcass characteristics, and the rise in dressing percentage is a direct result of increasing fatness with slaughter weight [35]. Thus, similar dressing percentage between experimental groups was due to unchanged fat contents in carcass in the current study. In addition, heavier striploin found in cows fed with higher straw levels and high rib found in younger cows were related to higher carcass weight. Taken together, the present results may not only be useful for further studies, but also for the sustainable and profitable beef production from cull cows.

In the current study, no significant significance, including the effects of age and diet and their interaction, was observed for IMF deposition. However, the mean value of IMF content in younger cows fed higher straw levels was 41.92% higher than in younger cows fed lower straw levels (31.0% vs. 21.9%). By contrast, the numerical difference in IMF content of older cows between diet groups was small (28.8% vs. 28.5%), which may indicate that IMF deposition is insensitive to the change of energy intake for older cows. It is known that the tenderness of beef from cull cows decreased with increasing age [1,2]. However, the shear force was unaffected by age in this study. A recent study [36] reported that the shear force of beef from cull cows was lower than that from heifers when fed with a high-energy ration for 150 d, which indicates that the high IMF content induced by long-term fattening could weaken the effect of connective tissue properties on tenderness. Drip loss, pressing loss as well as cooking loss can describe the water-holding capacity of beef and reflect different characteristics. Galli et al. (2008) [29] reported that cooking loss was unchanged with the increasing age of cull cows, which is consistent with our results. However, another recent study [37] showed that age altered the cooking loss of beef from cull cows and this influence depends on different beef cuts. In the current study, dietary treatment has no effect on the physical properties of beef except for cooking loss. The decreased cooking loss caused by high-level straw diet may lead to inferior juiciness.

Fatty acid composition can vary dramatically in beef depending on several factors, such as breed, sex, age, and diet [38]. Fruet et al. (2018) [39] reported that higher C18:1n9c and MUFAs proportion as well as lower C20:5n3c and SFAs proportion were found in the high concentrate diet treatment. Moreover, Wang et al. (2019) [27] reported that the proportions of C18:1n9c and MUFAs rose with dietary energy. In the current study, younger cows fed higher straw levels had higher C18:1n9c and MUFAs proportion as well as lower C20:5n3c and SFAs proportion than younger cows fed lower straw levels, which could be associated with the enhancement of energy intake. Although total TDN intake of two age classes both increased with straw levels, the effects of diet on the proportions of C15:0, C17:0, C18:1n9c, C20:5n3c, SFAs, and MUFAs were more pronounced in younger cows than in older cows. Several studies have demonstrated that the fatty acid profile was strongly affected by IMF content [40,41]. Thus, the interaction between age and diet for those fatty acids may partially come from their numerical difference in IMF content. Cho et al. (2013) [42] reported that the proportions of C18:3n3c, C18:3n6c, SFA, and n-3 PUFAs increased and MUFAs decreased in striploin as the cow age increased. However, in this study, differences were only detected for the slightly higher n-6/n-3 PUFAs ratio and greater C18:3n3c proportion in older cows. Reasons for the inconsistency in these results may be related to the differences in age classes.

Oxidative stress caused by the increased production of ROS or a decrease in antioxidant capacity, results in damage to biological macromolecules as well as disruption of normal metabolism and physiology [43]. Moreover, oxidative stress plays a key role in the pathogenesis of diverse diseases in cattle [44,45]. Antioxidases CAT, SOD, and glutathione peroxidase (GSH-Px) are part of enzymatic antioxidant systems that protect tissue components against oxidative stress caused by ROS. The concentration of MDA reflects the extent of lipid oxidation, which has a negative impact on meat freshness and quality, including undesirable off-flavor, toxic substances, and discoloration [46]. Protein carbonyl (PC) is a biomarker of protein oxidative damage, which could decrease the water-holding capacity and tenderness of the meat [47]. Halliwell (1994) [48] reported that animal age induced the oxidative damage to cellular macromolecules, such as lipids, proteins, and DNA. Moreover, Cho et al. (2015) [20] reported that oxidative deterioration on d2 post-slaughter was accelerated with older age, despite the increased activity of antioxidant enzymes. Enhanced activities of antioxidant enzymes during aging were the consequences of increased expression of the mRNA of antioxidant enzymes [49]. However, only the CAT of liver tissue was different between age groups in this study, and its activities were higher in younger cows than in older cows. In addition, the antioxidant parameters were unaffected by straw amounts except for SOD. Antioxidase CAT and SOD are coupled enzymes [50], but the activity of CAT and SOD in both liver and muscle tissues did not exhibit the same pattern in our results, most likely due to the additional effect of dietary stress in our study, including high energy intake and abnormal level of dietary forage. A high concentration of unsaturated fatty acids, particularly PUFAs, accelerates the lipid oxidation process [51]. In the current study, the unsaturation degree in meat was unaffected by animal age and straw amounts, which may be related to the similarity of MDA concentration in muscle between experimental groups. The results that T-AOC, ROS, PC and MDA remain unchanged indicate that the antioxidant status under high-concentration diets may be less affected by age and diet.

## 5. Conclusions

Both age and rice straw inclusion levels had impact on the growth performance and carcass traits of Yiling cull cow. Younger age increased ADG, BW gain, and feed conversion efficiency, while higher straw amounts increased DM intake and decreased NDF digestibility. The change of fatty acid composition caused by dietary rice straw levels was more pronounced in younger cows than in older cows. Younger cows fed higher rice straw levels had lower C15:0, C17:0, C20:5n3c, and SFAs proportion as well as higher C18:1n9c and MUFAs proportion compared to younger cows fed lower rice straw levels. Older age decreased the CAT activity of liver tissue, while higher straw amounts reduced the SOD activity of muscle tissue. Thus, selecting according to their age and providing adequate forage feed for Yiling cull cows would be of greater finishing benefit.

## Figures and Tables

**Table 1:** Ingredients and chemical compositions of the experimental diets.

Item ^1^	Concentrate	Rice Straw
Ingredient, % (on DM basis)		
Corn grain	53.2	−
Wheat bran	29.4	−
Wheat middling	15.0	−
Rapeseed meal	0.4	−
NaHCO_3_	1.0	−
NaCl	1.0	−
Chemical composition, % (on DM basis)		
OM	94.3	86.3
CP	12.0	5.1
EE	6.1	2.5
NDF	23.5	74.8
ADF	3.5	39.3
DM, % (on air-dry basis)	90.6	93.6
ME, Mcal/kg (on DM basis)	2.97	1.68

^1^ DM, dry matter; OM, organic matter; CP, crude protein; EE, ether extract; NDF, neutral detergent fiber; ADF, acid detergent fiber; ME, metabolizable energy.

**Table 2:** Effects of age and straw inclusion levels on growth performance of Yiling cull cows ^1^.

Items ^2^	YCC		OCC			*p*-Value		
	LRS	HRS	LRS	HRS	SEM	Age	Diet	Age × Diet
Intake (kg/d, on DM basis)								
Concentrate	3.73	4.44	3.44	3.54	0.32	0.10	0.23	0.36
DM	4.36	5.55	4.09	4.62	0.33	0.11	0.03	0.35
TDN	3.35	4.16	3.12	3.41	0.27	0.10	0.07	0.35
Apparent digestibility of nutrients (%)								
OM	70.9	64.3	70.6	62.9	3.56	0.82	0.06	0.88
CP	60.9	58.4	59.6	53.9	4.59	0.54	0.39	0.74
EE	85.1	81.2	78.5	75.9	3.43	0.11	0.36	0.85
NDF	62.4	54.8	64.6	52.9	3.35	0.98	0.01	0.55
Body weight (BW, kg)								
Intial BW	330	333	331	329	9.4	0.85	−	−
Final BW	390	429	348	362	13.8	0.002	0.08	0.36
Total BW gain	55.2	90.4	22.1	33.0	13.7	0.006	0.12	0.39
ADG (g/d)	182.0	281.8	74.4	113.8	34.7	0.002	0.07	0.41
G:F (g/kg)	42.6	49.9	14.0	23.4	7.2	0.005	0.28	0.89

^1^ YCC, younger cull cows; OCC, older cull cows; LRS, providing 0.7 kg/d rice straw per head; HRS, providing 1.2 kg/d rice straw per head. ^2^ DM, dry matter; TDN, total digestible nutrients; TDN (kg) = ME (Mcal)/3.62; OM, organic matter; CP, crude protein; EE, ether extract; NDF, neutral detergent fiber; ADG, average daily gain; G:F, gain:feed intake.

**Table 3:** Effects of age and straw inclusion levels on carcass characteristics of Yiling cull cows ^1^.

Items ^2^	YCC		OCC		SEM	*p*-Value		
	LRS	HRS	LRS	HRS		Age	Diet	Age × Diet
CCW (kg)	218	246	187	205	9.3	0.002	0.03	0.57
Dressing percentage (%)	57.2	57.2	55.1	56.6	1.0	0.17	0.43	0.45
Carcass composition (%)								
Meat	60.9	61.9	59.6	60.0	1.94	0.42	0.74	0.88
Fat	25.5	26.4	23.6	22.9	1.90	0.35	0.42	0.71
Meat to fat ratio	2.51	2.38	2.30	2.10	0.23	0.30	0.49	0.88
LM area (cm^2^)								
Between 6th and 7th ribs	29.6	31.1	25.7	26.2	2.86	0.15	0.73	0.86
Between 12th and 13th ribs	64.3	71.3	56.8	63.6	3.92	0.07	0.10	0.98
Top grade cuts weight (kg)								
High rib	8.85	11.34	8.64	8.17	0.72	0.04	0.19	0.06
Ribeye	7.10	8.00	6.55	7.14	0.74	0.37	0.34	0.85
Striploin	5.83	7.07	5.15	6.52	0.47	0.21	0.02	0.90
Tenderloin	2.68	3.10	2.44	2.45	0.18	0.02	0.25	0.27

^1^ YCC, younger cull cows; OCC, older cull cows; LRS, providing 0.7 kg/d rice straw per head; HRS, providing 1.2 kg/d rice straw per head. ^2^ CCW, Cold carcass weight.

**Table 4:** Effects of age and straw inclusion levels on meat quality of LM ^1^ from Yiling cull cows ^2^.

Items ^3^	YCC		OCC			*p*-Value		
	LRS	HRS	LRS	HRS	SEM	Age	Diet	Age × Diet
pH, day 0	6.22	6.26	6.11	6.37	0.14	1.00	0.33	0.47
pH, day 7	5.66	5.62	5.77	5.74	0.11	0.32	0.74	0.99
DM (%)	30.9	34.3	34.2	32.2	1.43	0.68	0.61	0.08
IMF (%)	21.9	31.0	28.5	28.8	3.96	0.60	0.25	0.28
CP (%)	72.5	62.5	66.3	66.2	4.40	0.78	0.27	0.28
WBSF (N)	26.9	31.6	31.2	29.7	3.23	0.71	0.63	0.36
Drip loss (%)	6.56	5.51	6.45	5.87	1.33	0.93	0.56	0.86
Pressing loss (%)	30.5	32.4	31.4	31.3	1.60	0.98	0.57	0.53
Cooking loss (%)	26.9	24.5	27.5	25.2	0.86	0.48	0.02	1.00

^1^ LM, *longissimus dorsi* muscle. ^2^ YCC, younger cull cows; OCC, older cull cows; LRS, providing 0.7 kg/d rice strawper head; HRS, providing 1.2 kg/d rice straw per head. ^3^ DM, dry matter; IMF, intramuscular fat, based on DM basis; CP, crude protein, based on DM basis; WBSF, Warner-Bratzler shear force.

**Table 5:** Effects of age and straw inclusion levels on fatty acid profile (%, of total fatty acids) of LM ^1^ from Yiling cull cows ^2^.

Items ^3^	YCC		OCC			*p*-Value		
	LRS	HRS	LRS	HRS	SEM	Age	Diet	Age × Diet
Saturated								
C14:0	3.04	3.28	3.28	3.66	0.29	0.31	0.30	0.81
C15:0	0.29 a	0.21 b	0.21 b	0.23 b	0.016	0.13	0.10	0.006
C16:0	28.95	26.48	26.77	28.64	0.94	0.99	0.75	0.04
C17:0	0.80 a	0.59 b	0.59 b	0.62 b	0.036	0.03	0.03	0.006
C18:0	12.25	10.08	11.22	10.80	0.79	0.85	0.12	0.29
C20:0	0.11	0.10	0.10	0.10	0.006	0.67	0.18	0.48
C21:0	0.05	0.05	0.06	0.04	0.008	0.93	0.10	0.12
C22:0	0.06	0.03	0.04	0.04	0.010	0.70	0.16	0.32
Monounsaturated								
C14:1n5c	1.19	1.44	1.36	1.35	0.16	0.80	0.47	0.42
C15:1n5c	0.22	0.15	0.14	0.15	0.045	0.34	0.52	0.40
C16:1n7c	4.01	4.84	4.87	4.98	0.37	0.20	0.22	0.35
C17:1n7c	0.69	0.62	0.57	0.61	0.046	0.16	0.72	0.27
C18:1n9t	0.47	0.48	0.74	0.38	0.095	0.39	0.08	0.07
C18:1n9c	43.66 b	48.19 a	46.33 ab	44.19 ab	1.35	0.63	0.39	0.03
C22:1n9c	0.82	0.38	0.45	0.53	0.14	0.48	0.22	0.09
C24:1n9c	0.09	0.05	0.05	0.07	0.017	0.57	0.65	0.15
Polyunsaturated								
C18:2n6c	2.49	2.33	2.51	2.89	0.37	0.44	0.76	0.47
C18:3n6c	0.08	0.06	0.06	0.07	0.014	0.88	0.56	0.36
C18:3n3c	0.19	0.25	0.23	0.23	0.015	0.67	0.04	0.08
C20:2n6c	0.04	0.05	0.07	0.04	0.011	0.48	0.46	0.16
C20:3n6c	0.22	0.17	0.18	0.17	0.046	0.68	0.52	0.69
C20:3n3c	0.02	0.02	0.02	0.02	0.003	0.77	0.45	0.30
C20:4n6c	0.03	0.02	0.02	0.03	0.005	0.48	0.66	0.09
C20:5n3c	0.06a	0.03b	0.03b	0.05ab	0.008	0.36	0.51	0.02
C22:6n3c	0.16	0.10	0.09	0.11	0.027	0.23	0.48	0.11
ΣSFA	45.57 a	40.82 b	42.28 ab	44.14 ab	1.46	0.99	0.34	0.04
ΣMUFA	51.14 b	56.15 a	54.52 ab	52.25 b	1.31	0.85	0.32	0.02
ΣPUFA	3.29	3.03	3.20	3.61	0.45	0.60	0.87	0.47
ΣPUFA/ΣMUFA	0.06	0.05	0.06	0.07	0.009	0.54	0.92	0.24
Σn-6 PUFA	2.85	2.63	2.83	3.20	0.41	0.52	0.87	0.49
Σn-3 PUFA	0.44	0.40	0.36	0.41	0.042	0.44	0.89	0.32
Σn-6/Σn-3 PUFA	6.50	6.50	7.87	7.57	0.63	0.07	0.81	0.81

ab Means followed by different letters in the same row are significant at the *p* < 0.05. ^1^ LM, *longissimus dorsi* muscle.^2^ YCC, younger cull cows; OCC, older cull cows; LRS, providing 0.7 kg/d rice straw per head; HRS, providing 1.2 kg/d rice straw per head. ^3^ ΣSFA, sum of saturated fatty acids (C14:0, C15:0, C16:0, C17:0, C18:0, C20:0, C21:0, C22:0); ΣMUFA, sum of monounsaturated fatty acids (C14:1n5c, C15:1n5c, C16:1n7c, C17:1n7c, C18:1n9t, C18:1n9c, C22:1n9c, C24:1n9c); ΣPUFA, sum of polyunsaturated fatty acids (i.e., sum of Σn-6 and Σn-3); Σn-6, sum of C18:2n6c, C18:3n6c, C20:2n6c, C20:3n6c, C20:4n6c; Σn-3, sum of C18:3n3c, C20:3n3c, C20:5n3c, C22:6n3c.

**Table 6:** Effects of age and straw inclusion levels on antioxidant status of tissues from Yiling cull cows ^1^.

Items ^2^	YCC		OCC		SEM	*p*-Value		
	LRS	HRS	LRS	HRS		Age	Diet	Age × Diet
T-AOC (umol Trolox/Mgprot)								
Liver	0.28	0.26	0.26	0.21	0.02	0.11	0.15	0.61
Muscle	1.17	1.25	1.12	1.18	0.10	0.55	0.48	0.91
CAT (U/mgprot)								
Liver	4.78	3.85	3.29	3.28	0.45	0.045	0.33	0.34
Muscle	3.16	4.59	4.28	6.53	1.14	0.20	0.13	0.73
SOD (U/mgprot)								
Liver	396	382	370	380	17.0	0.42	0.92	0.50
Muscle	1365	1189	1315	1178	52.5	0.57	0.01	0.71
ROS (U/mgprot)								
Liver	13.2	12.9	13.6	13.2	0.62	0.57	0.63	0.99
Muscle	122.7	116.4	124.3	119.7	5.07	0.63	0.30	0.87
MDA (nmol/mgprot)								
Liver	2.50	2.27	2.36	2.43	0.13	0.93	0.53	0.25
Muscle	17.2	16.7	15.9	16.5	0.99	0.43	0.97	0.59
PC (nmol/mgprot)								
Liver	12.2	11.4	10.2	11.4	1.23	0.43	0.87	0.43
Muscle	53.4	66.9	57.2	45.3	7.37	0.25	0.92	0.11

^1^ YCC, younger cull cows; OCC, older cull cows; LRS, providing 0.7 kg/d rice straw per head; HRS, providing 1.2 kg/d rice straw per head. ^2^ T-AOC: total antioxidative capacity; CAT, catalase; SOD: superoxide dismutase; ROS, reactive oxygen species. MDA: malondialdehyde; PC: protein carbonyl.
